# Supplementary figures and images for: WRNIP1 prevents transcription-associated genomic instability
Source: eLife. 2024 Mar 15;12:RP89981. doi: 10.7554/eLife.89981 (PMC10942783; doi:10.7554/eLife.89981)

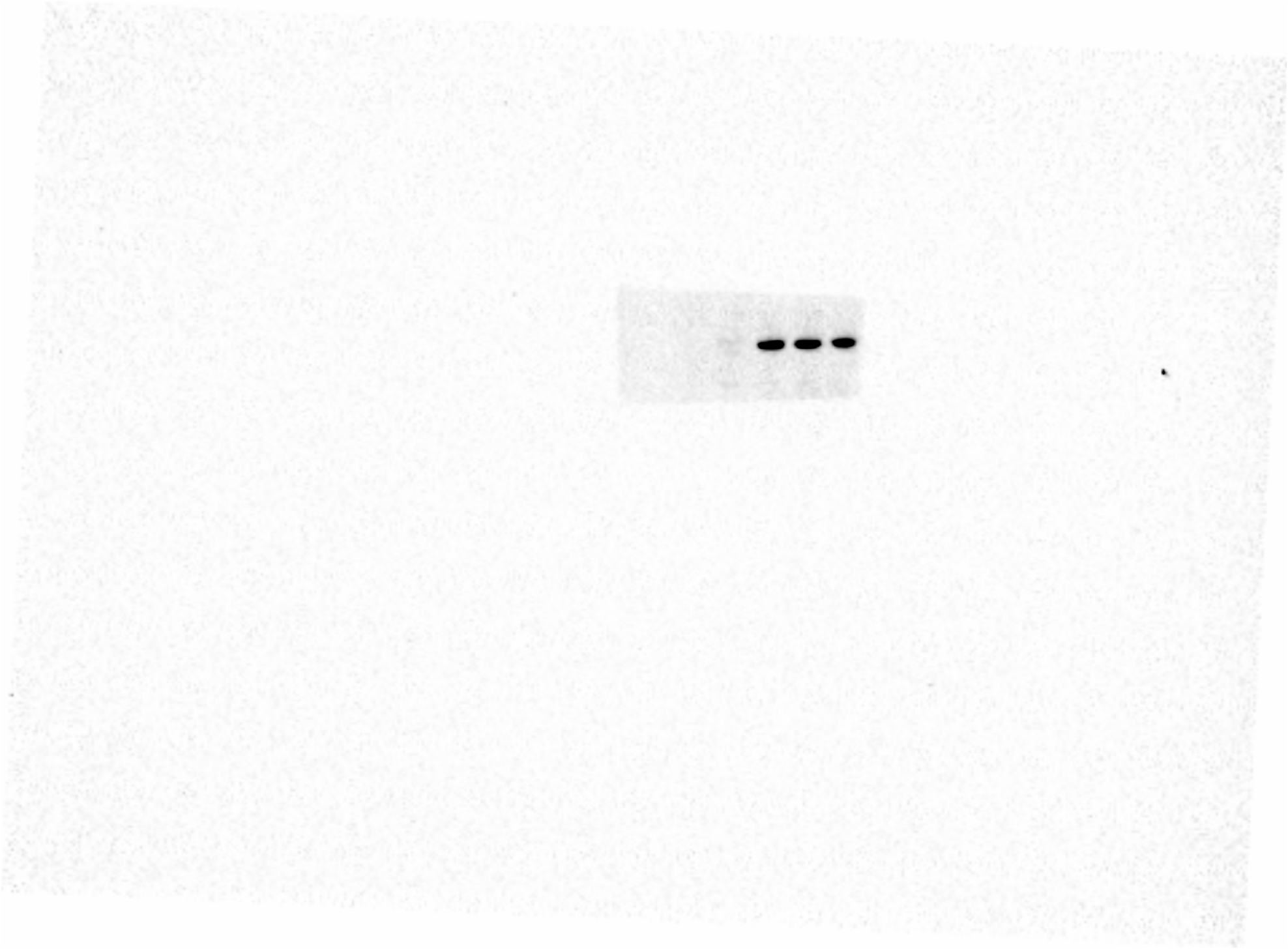

Supplement: Figure 1—source data 1. [file elife-89981-fig1-data1.zip › Figure 1- source data 1/Fig 1_FLAG_sourceblot.tif]

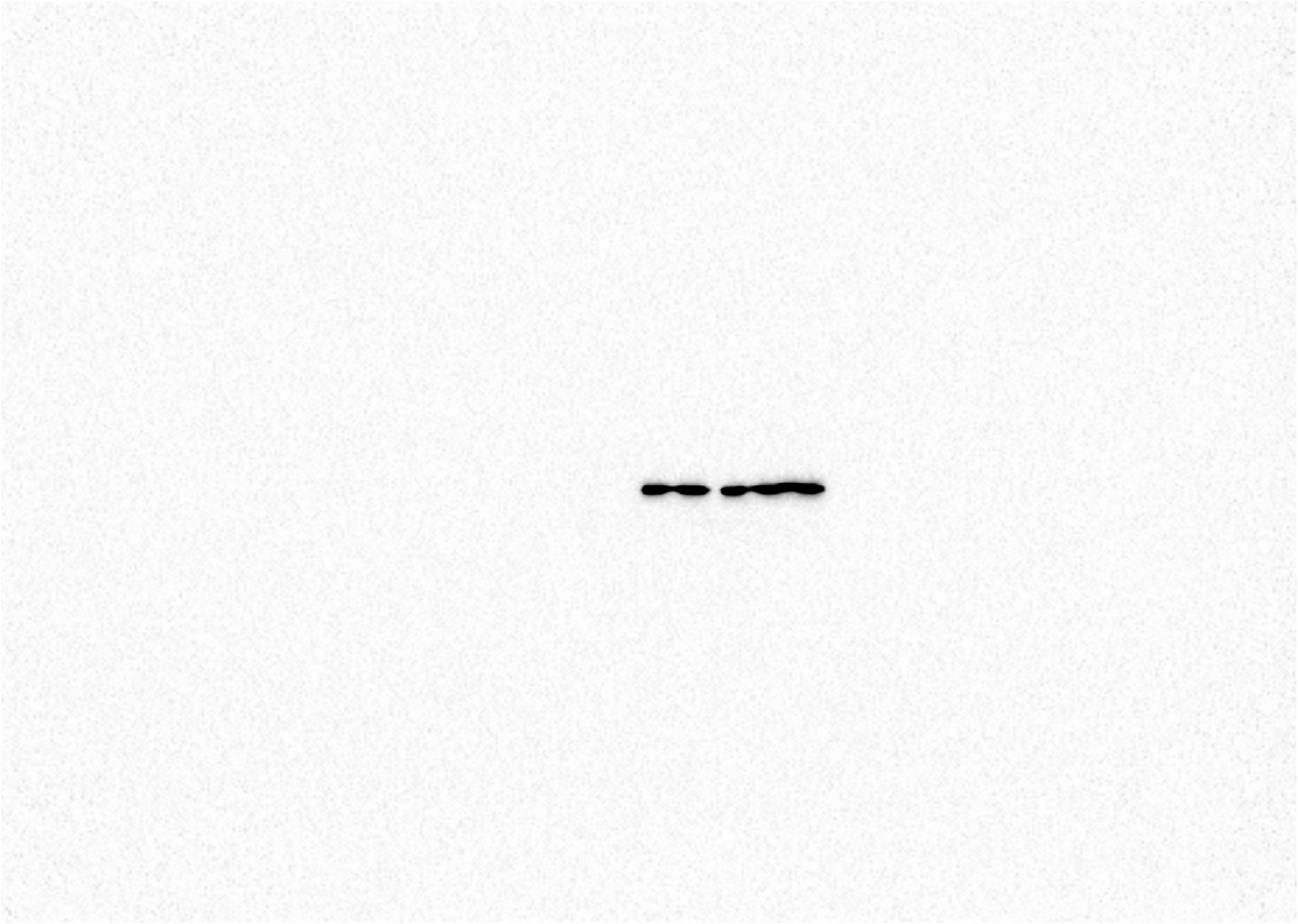

Supplement: Figure 1—source data 1. [file elife-89981-fig1-data1.zip › Figure 1- source data 1/Fig 1_GAPDH_sourceblot.tif]

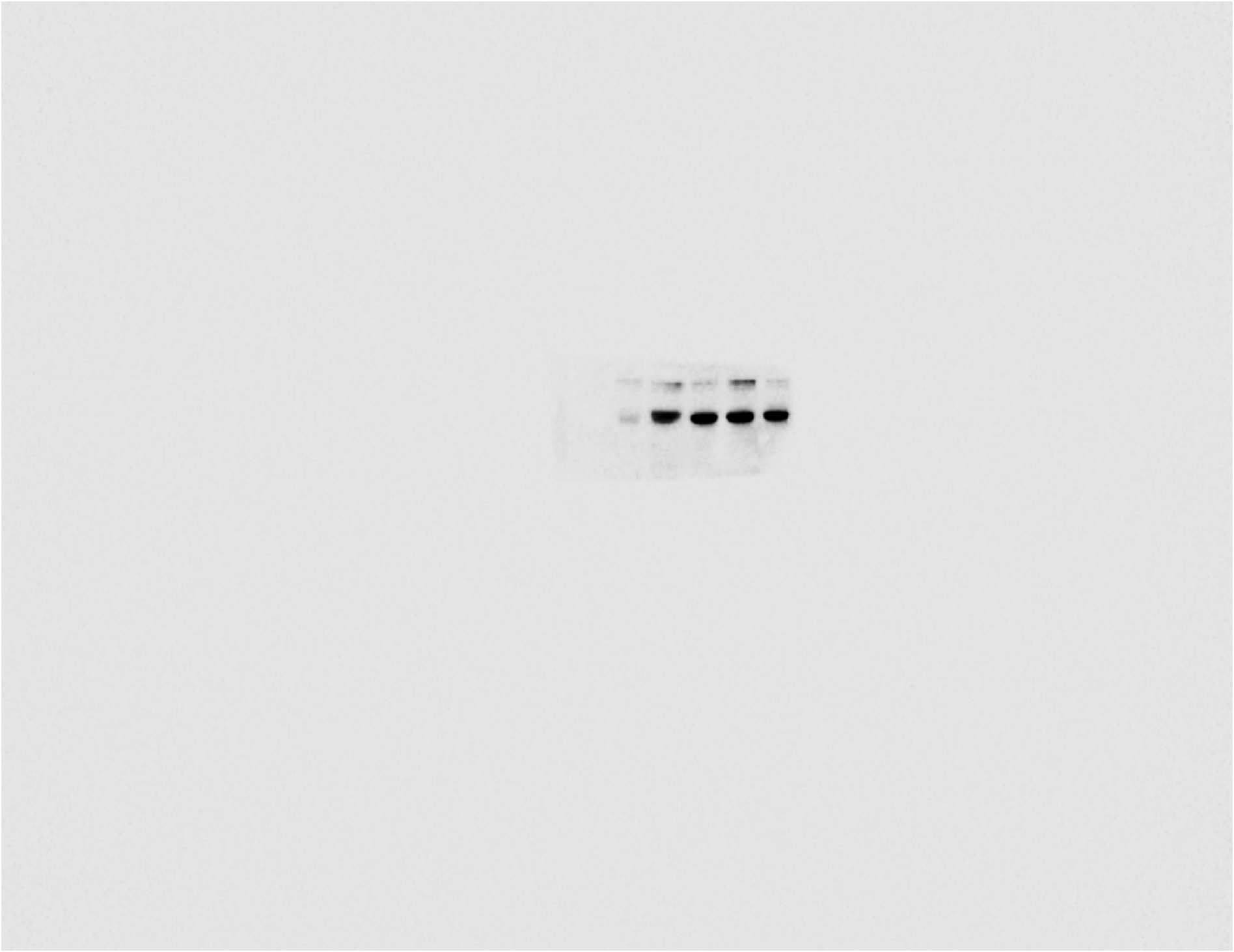

Supplement: Figure 1—source data 1. [file elife-89981-fig1-data1.zip › Figure 1- source data 1/Fig 1_WRNIP1_sourceblot.tif]

Figure 1 – Source data 1

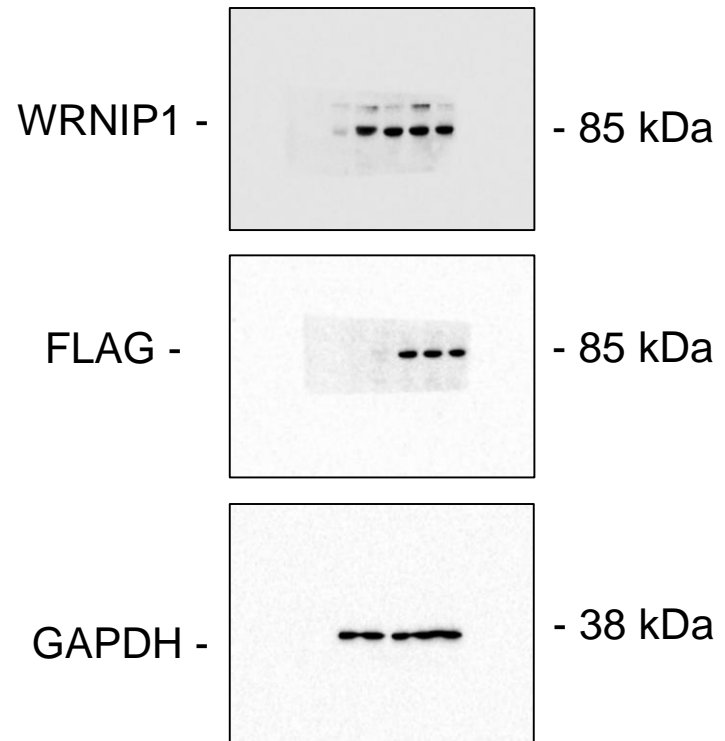

Supplement: Figure 1—source data 1. [file elife-89981-fig1-data1.zip › Figure 1- source data 1/Figure 1- source data 1.pdf]

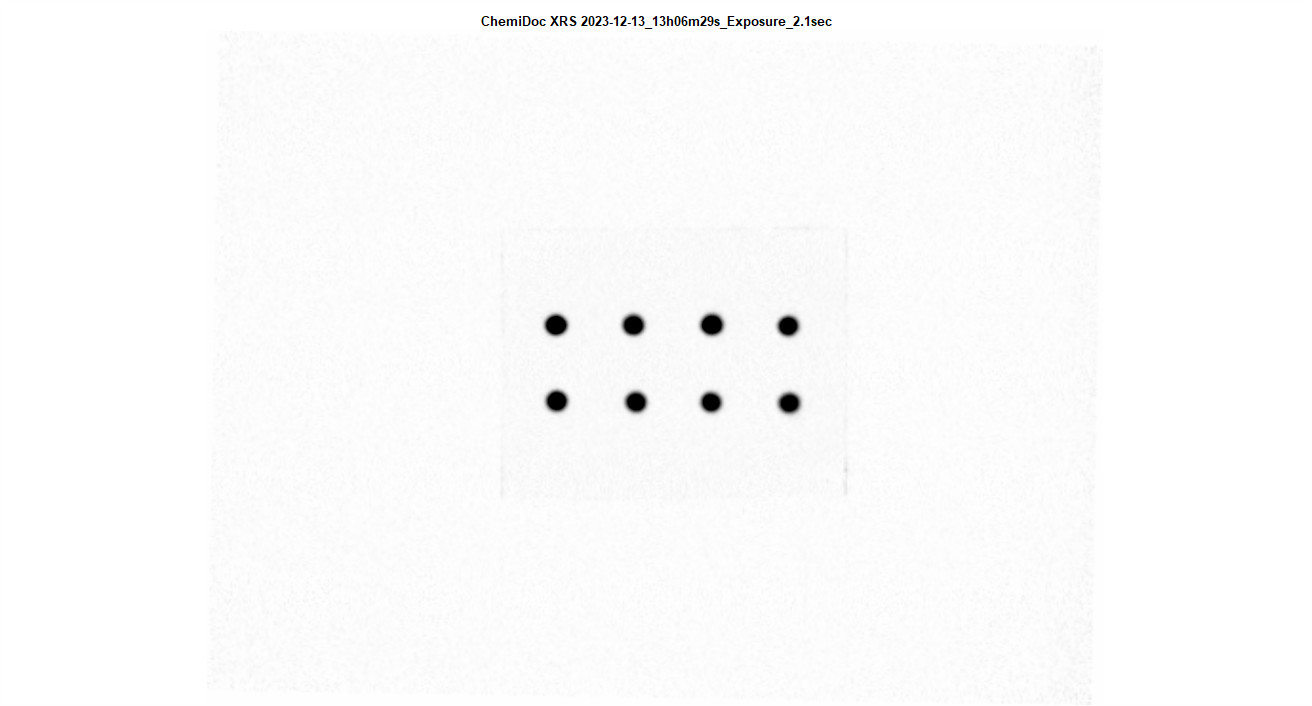

Supplement: Figure 2—source data 1. [file elife-89981-fig2-data1.zip › Figure 2- source data 1/Fig 2_dsDNA_sourceblot.tif]

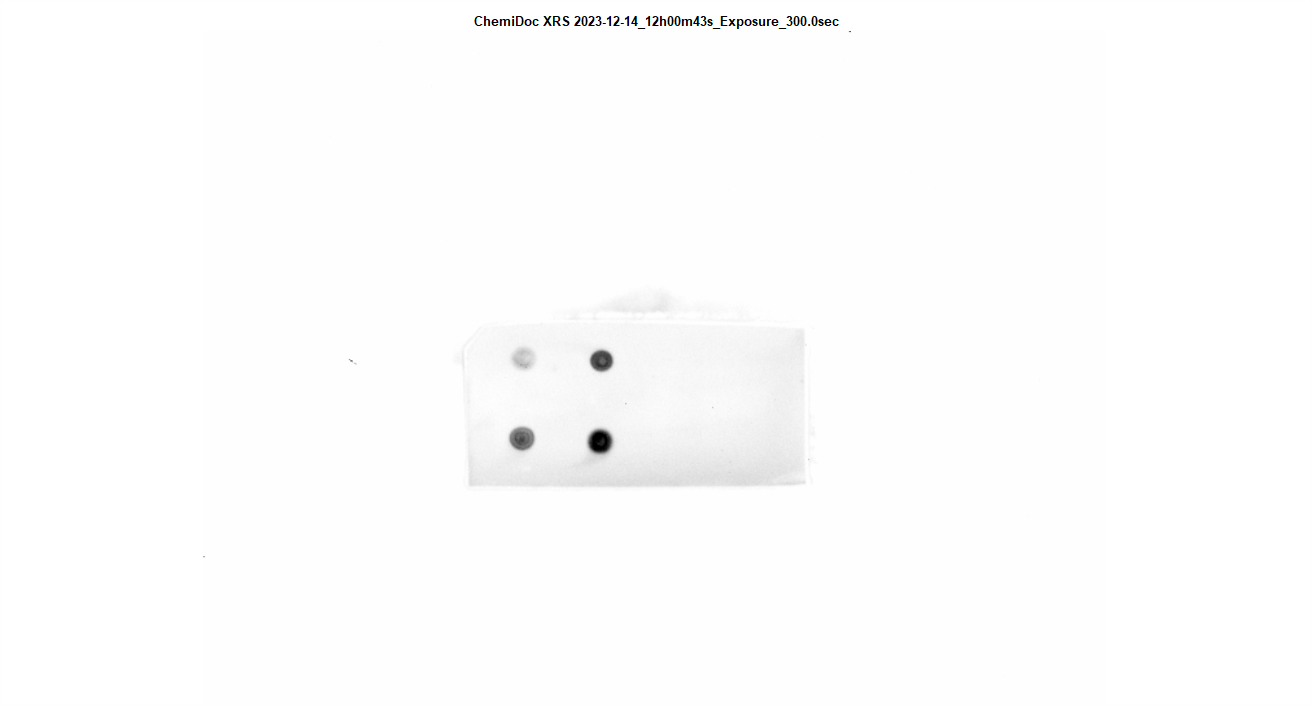

Supplement: Figure 2—source data 1. [file elife-89981-fig2-data1.zip › Figure 2- source data 1/Fig 2_S9.6_sourceblot.tif]

Figure 2 – Source data 1

IB: S9.6

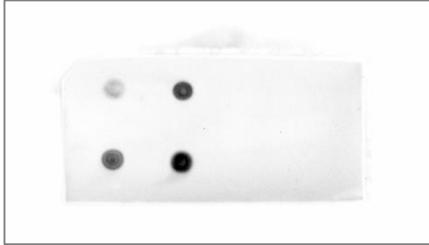

IB: dsDNA

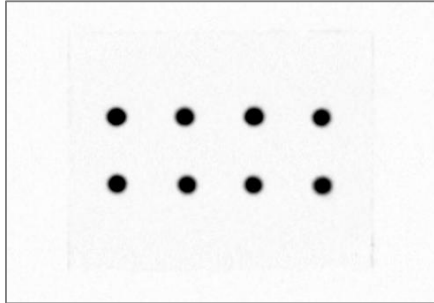

Supplement: Figure 2—source data 1. [file elife-89981-fig2-data1.zip › Figure 2- source data 1/Figure 2- source data 1.pdf]

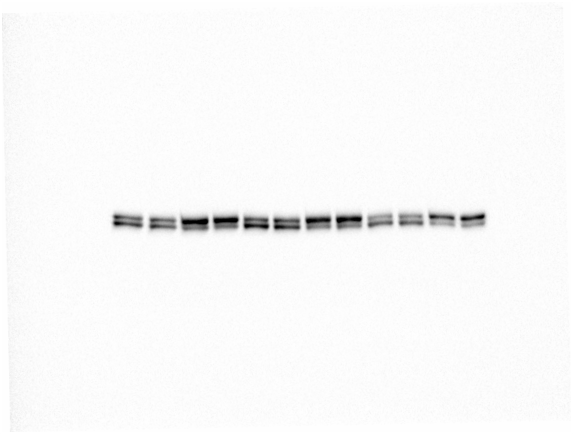

Supplement: Figure 7—source data 1. [file elife-89981-fig7-data1.zip › Figure 7- source data 1/Fig 7_FANCD2_sourceblot.tif]

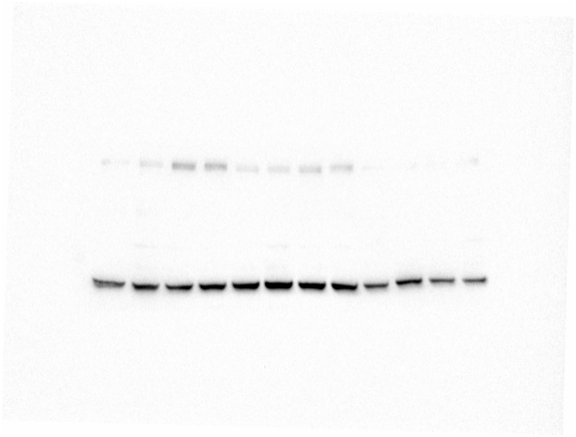

Supplement: Figure 7—source data 1. [file elife-89981-fig7-data1.zip › Figure 7- source data 1/Fig 7_LAMINB1_sourceblot.tif]

Figure 7 – Source data 1

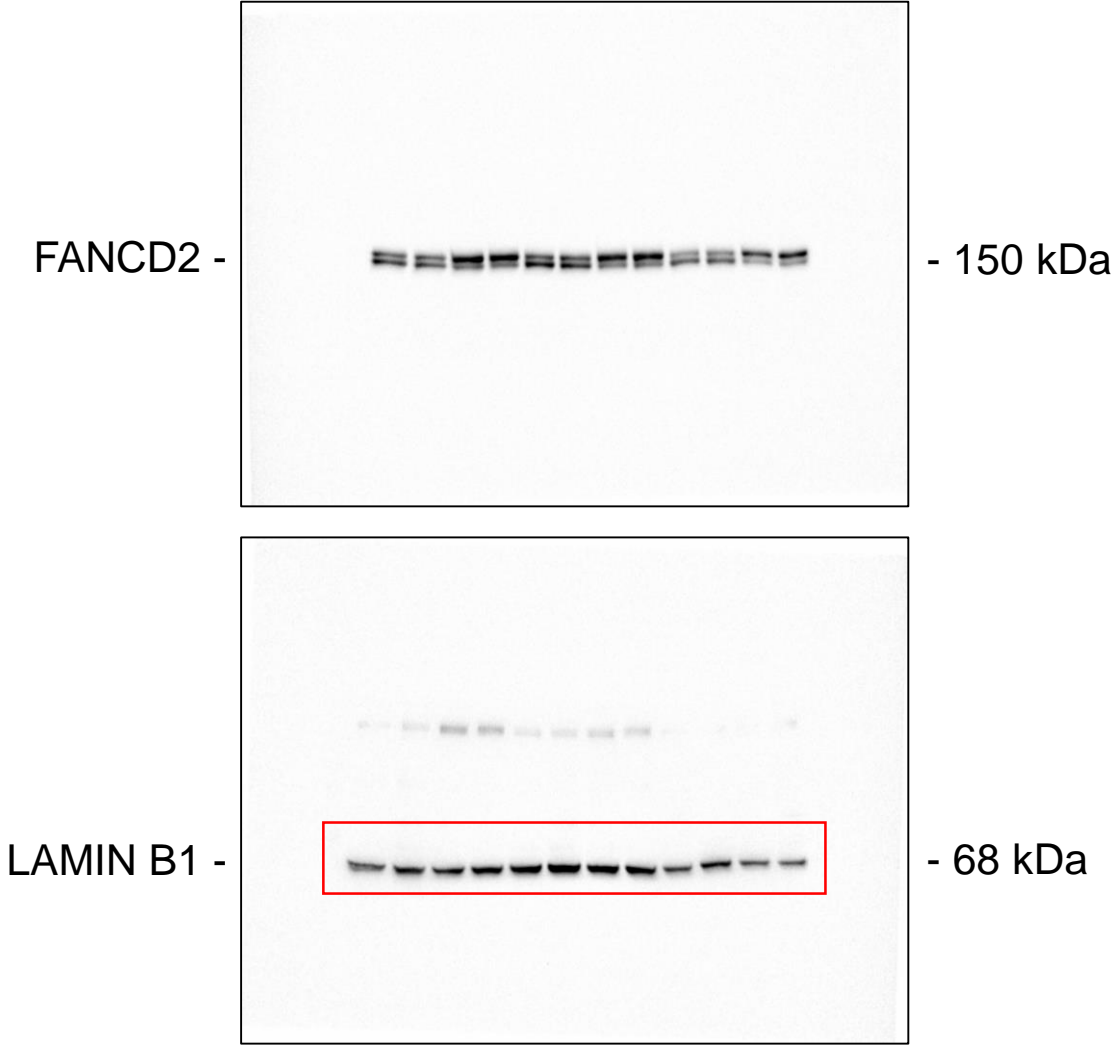

Supplement: Figure 7—source data 1. [file elife-89981-fig7-data1.zip › Figure 7- source data 1/Figure 7- source data 1.pdf]

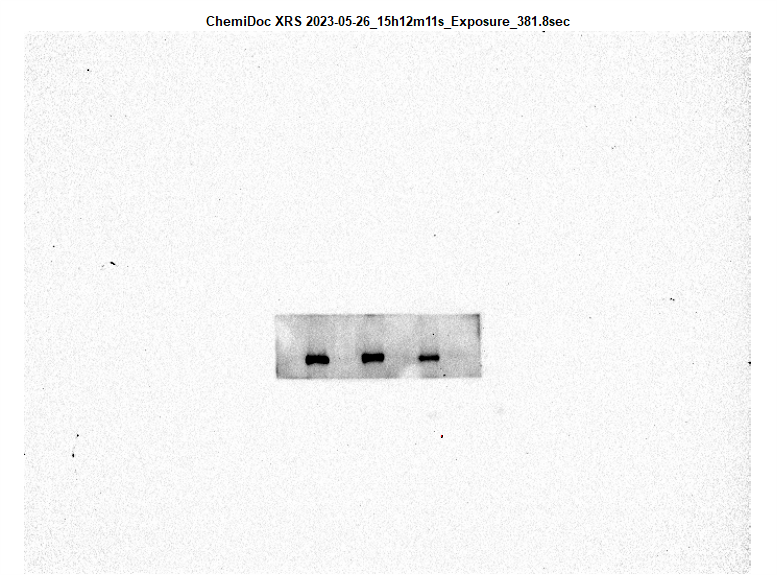

Supplement: Figure 7—source data 2. [file elife-89981-fig7-data2.zip › Figure 7- source data 2/Fig 7_2_FANCD2_sourceblot.tif]

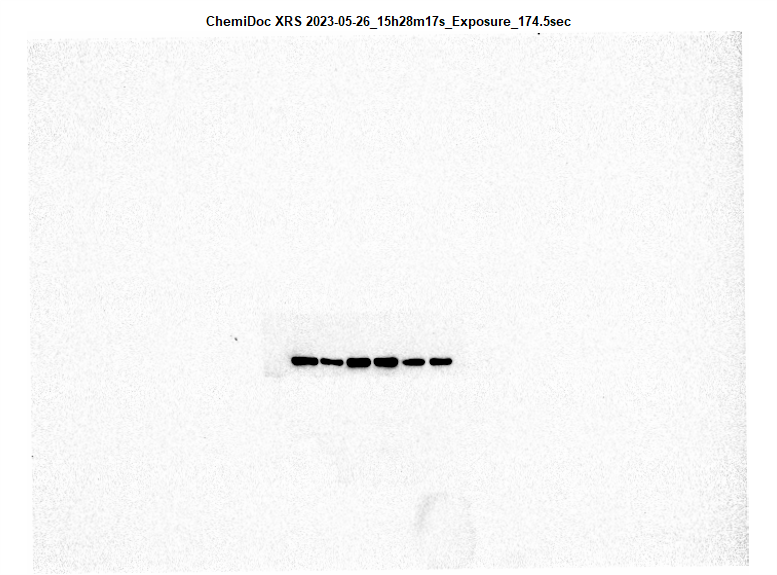

Supplement: Figure 7—source data 2. [file elife-89981-fig7-data2.zip › Figure 7- source data 2/Fig 7_2_LAMINB1_sourceblot.tif]

Figure 7 – Source data 2

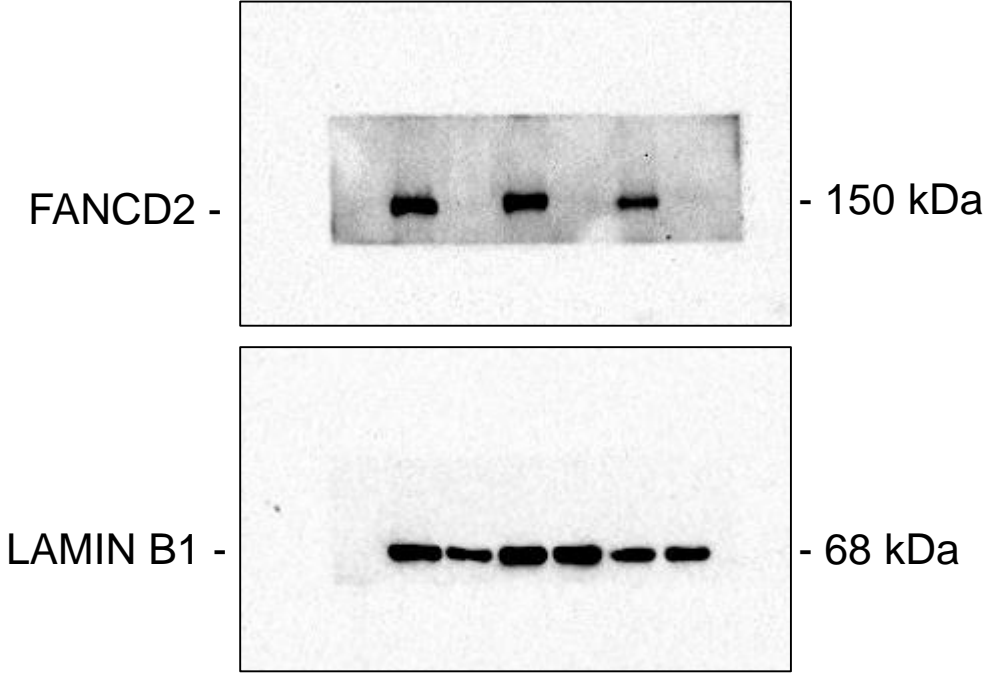

Supplement: Figure 7—source data 2. [file elife-89981-fig7-data2.zip › Figure 7- source data 2/Figure 7- source data 2.pdf]

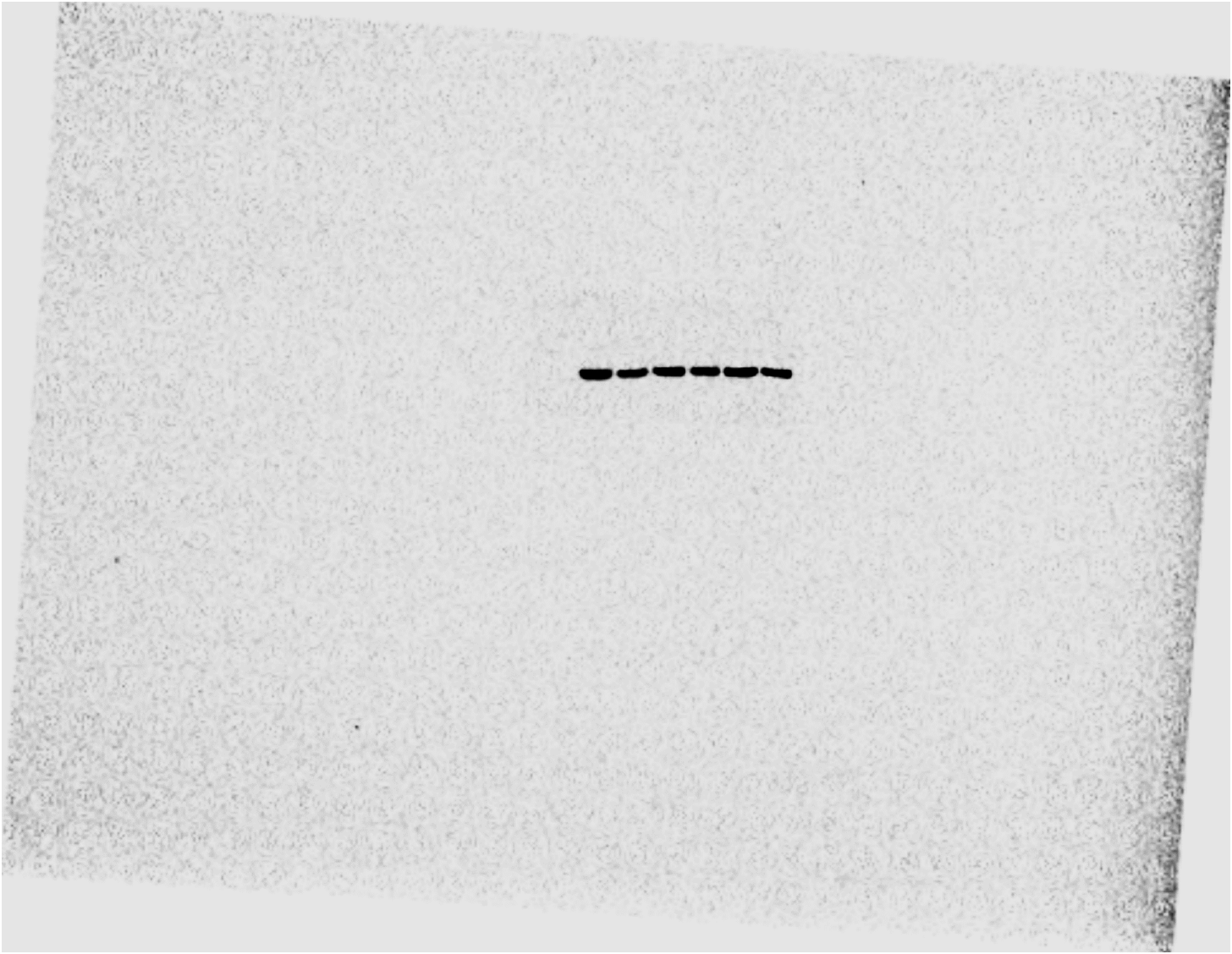

Supplement: Figure 7—figure supplement 2—source data 1. [file elife-89981-fig7-figsupp2-data1.zip › Figure 7-figure supplement 2-Source data 1/Fig 7_FIGSUPP2_LAMINB1_sourceblot.tif]

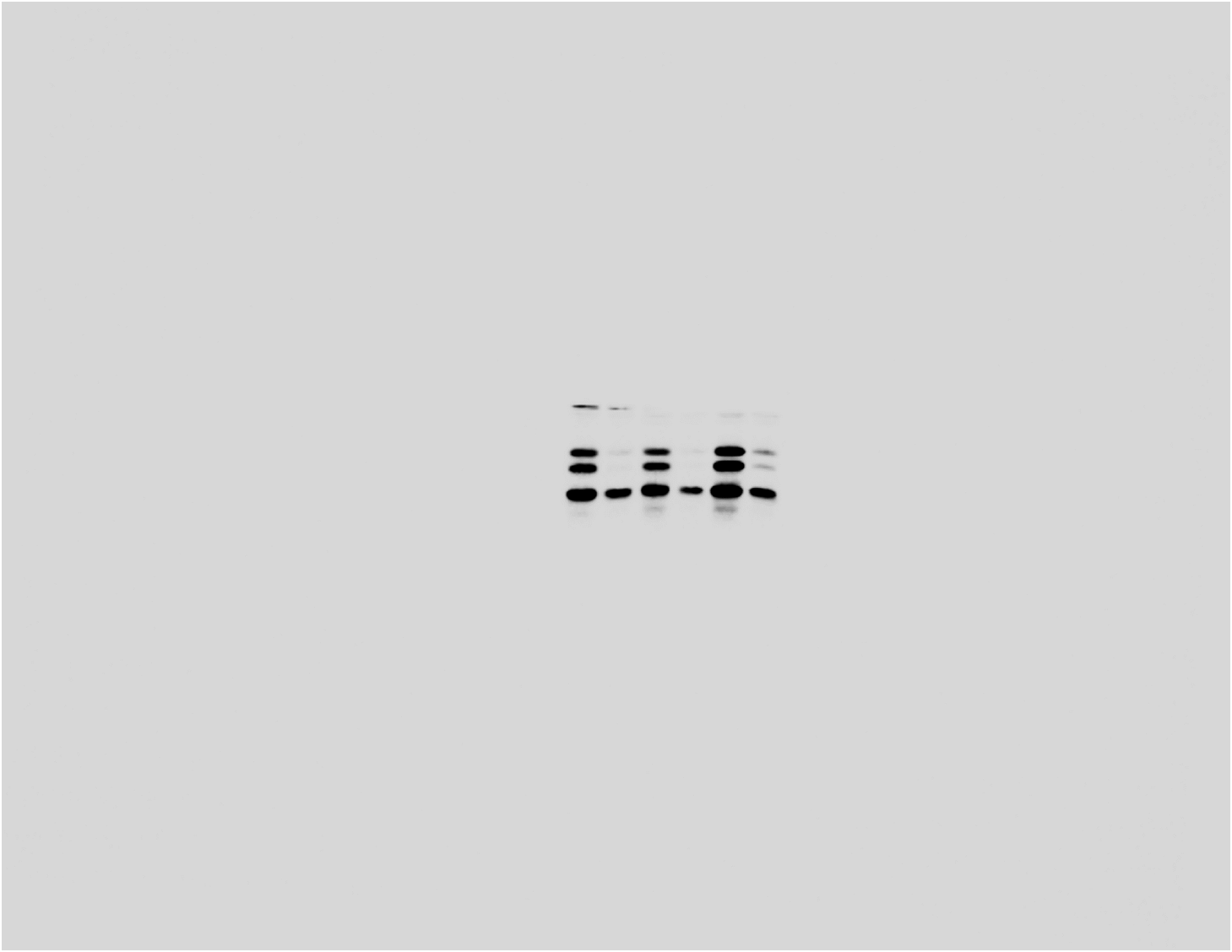

Supplement: Figure 7—figure supplement 2—source data 1. [file elife-89981-fig7-figsupp2-data1.zip › Figure 7-figure supplement 2-Source data 1/Fig 7_FIGSUPP2_RAD18_sourceblot.tif]

Figure 7 - figure supplement 2– Source data 1

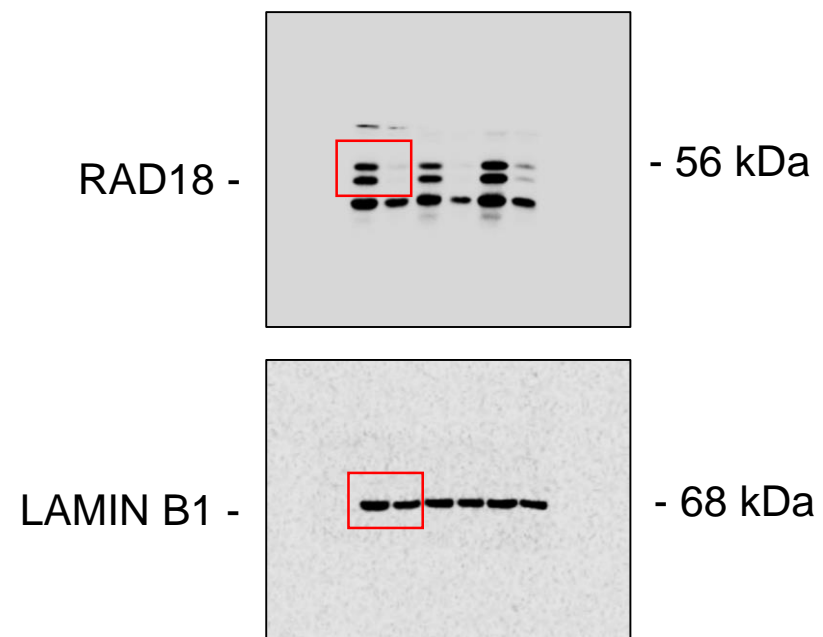

Supplement: Figure 7—figure supplement 2—source data 1. [file elife-89981-fig7-figsupp2-data1.zip › Figure 7-figure supplement 2-Source data 1/Figure 7 - figure supplement 2-Source data 1.pdf]
